# The Southern Medical Record

**Published:** 1873-02

**Authors:** 


### PART III.

### EDITORIAL AND MISCELLANEOUS.

THE SOUTHERN MEDICAL RECORD.—The objects in the publication of the RECORD are: First, to lay before the profession the truths of medical science, as they are being developed from month to month, in a condensed form for practical use. Second, to furnish an independent medium through which our medical brethren, especially in the South, may communicate gems of thought and experience now lost to medical literature. Third, to bring before our readers all the new improvements in therapeutics and instruments. Fourth, to call attention to standard works of authors, that they may read and be instructed thereby.

With these objects in view, we are satisfied our readers will extend to us and to the RECORD their aid and influence. We solicit original articles, reports of societies, clinics, news, etc., of interest to medical readers. Articles should be received by the 15th of one month to insure publication in the next. Country physicians are especially invited to communicate their experience through the RECORD.

